# A Cooperative Lightweight Translation Algorithm Combined with Sparse-ReLU

**DOI:** 10.1155/2022/4398839

**Published:** 2022-05-28

**Authors:** Xintao Xu, Yi Liu, Gang Chen, Junbin Ye, Zhigang Li, Huaxiang Lu

**Affiliations:** ^1^School of Microelectronics, University of Science and Technology of China, Hefei, China; ^2^Institute of Semiconductors, Chinese Academy of Sciences, Beijing, China; ^3^Materials and Optoelectronics Research Center, University of Chinese Academy of Sciences, Beijing, China; ^4^College of Microelectronics, University of Chinese Academy of Sciences, Beijing, China; ^5^Semiconductor Neural Network Intelligent Perception and Computing Technology Beijing Key Laboratory, Beijing, China

## Abstract

In the field of natural language processing (NLP), machine translation algorithm based on Transformer is challenging to deploy on hardware due to a large number of parameters and low parametric sparsity of the network weights. Meanwhile, the accuracy of lightweight machine translation networks also needs to be improved. To solve this problem, we first design a new activation function, Sparse-ReLU, to improve the parametric sparsity of weights and feature maps, which facilitates hardware deployment. Secondly, we design a novel cooperative processing scheme with CNN and Transformer and use Sparse-ReLU to improve the accuracy of the translation algorithm. Experimental results show that our method, which combines Transformer and CNN with the Sparse-ReLU, achieves a 2.32% BLEU improvement in prediction accuracy and reduces the number of parameters of the model by 23%, and the sparsity of the inference model increases by more than 50%.

## 1. Introduction

Machine translation, an essential branch of computational linguistics, is a process of translating source language into the target language by computer. Translation has extremely high requirements for translators, and at the same time, there is a lack of professional translators, so machine translation has made significant progress in international exchanges [1]. In recent years, deep learning technology has developed rapidly. Researchers have introduced neural network into language model, which can better process the representation of common and rare words. For example, a recurrent neural network (RNN) can adapt to any sentence length and process the context recurrently to get the final result. Transformer applies the attention mechanism to machine translation and has better translation quality than traditional methods.

Compared with traditional statistical machine translation, which requires elaborate features, the flexibility of existing machine translation based on neural networks is greatly improved. Methods based on RNN and its derived models such as GRU and LSTM need to learn the long-distance dependencies of each input word vector. The principle is to use the embedding layer to map sentences to the embedding space and then use the hidden layer to compute the knowledge obtained in the previous step. As multiple hidden layers compute sequentially, calculations within a single hidden layer are executed sequentially and cannot be carried out in parallel. Different from the scheme of the RNN model that continuously accumulates input information, the Transformer network uses the Encoder-Decoder structure.

Because of its stacking self-attention layer and point-by-point full connection layer, the recursive structure in RNN is eliminated, and the network based on Transformer has the advantage of high parallelism. Transformer offers significant improvements to machine translation, but at the cost of a large number of parameters. The number of BERT large parameters is 334M, the number of BERT base parameters is 109M, and the number of IB-BERT large parameters is 293M. Due to its large number of parameters and low sparsity of parameters, it is generally applied to the server side, and there is no suitable edge side Transformer algorithm. The existing RNN model must wait for all previous input processing to be completed before processing the next input, which is a bottleneck when processing long sequences. The number of operations required by the RNN model to correlate information from two arbitrary input or output positions increases as the position distance increases. This makes extracting complicated dependencies between faraway positions more difficult. Therefore, RNN is difficult to parallel, which is not conducive to hardware acceleration, and the translation effect is not ideal. On the other hand, the hardware-implementation-friendly CNN can not effectively process location information and is not effective in machine translation tasks when applied alone.

It is an effective method for hardware deployment of the neural network to reduce parameter storage and transmission amount and reduce the dependence on hardware data transmission bandwidth by using the compressed sparse matrix method. And it has little influence on algorithm accuracy. The premise of this scheme is that the sparsity of algorithm parameters is high enough. The weight parameters of the Transformer have not been optimized for sparsity, which is difficult to be applied to hardware accelerated by a compressed sparse matrix [2, 3]. The traditional ReLU activation function adopted by Transformer models such as [4] does not improve the sparsity of the neural network algorithm to the maximum extent. An appropriate activation function is an important measure to improve network performance and reduce the number of network parameters.

The hardware deployment machine translation algorithm must meet the requirements of lightweight and maintain high-precision translation results. At the same time, in order to further reduce the difficulty of deployment on edge devices, the algorithm optimization must improve the sparsity of weight parameters, so as to use the sparse matrix compression method for hardware deployment. To solve the above problems, this research proposes a new activation function that can improve the algorithm accuracy and parameter sparsity at the same time and designs a cooperative machine translation algorithm combining CNN to extract local features and Transformer to process sequence information. Our method combined with Sparse-ReLU improved the BLEU score of the algorithm to 35.24, increased the sparsity by more than 150%, and controlled the total number of parameters within 38M. Our main contributions are as follows:

1. A new activation function, Sparse-ReLU, is proposed and applied to the machine translation model. The BLEU score of the IWSLT14 German-English translation task is enhanced from 34.29 to 35.16 by using the model whose parameter scale is 36.42 M. The number of parameters has been reduced, and more than 50% of sparsity has grown. Meanwhile, Sparse-ReLU can improve the translation effect.
2. A Transformer structure with low number of parameters is proposed, which only uses three attention heads and a 7-layer encoder and decoder. The number of parameters of this structure is only 36.42 M, which solves the problem that Transformer is too large to be deployed on the hardware.
3. A CNN structure for machine translation tasks is proposed and combined with a Transformer to optimize the network. The number of parameters of the overall algorithm is 37.99 M. The BLEU score is increased from 35.16 to 35.24.

### 1.1. Related Works

The machine translation algorithm based on the neural network generally adopts the Encoder-Decoder model to deal with the machine translation task [5]. The encoder takes the source sentence as input and calculates a real expression value. The decoder inputs the real expression value and generates the target translation. CNN, RNN, and Transformer are the classical algorithms for constructing the Encoder-Decoder structure.

Machine translation jobs can be processed serially using an approach based on RNN and its derivatives LSTM [6, 7] and GRU [8]. It has the advantage of high extraction ability in processing series information. For example, the RNN-based algorithm [9, 10] generates dynamic context representation through its Encoder-Decoder architecture based on attention mechanism. Research [11] creates target phrases with fixed source statement representation. To make the RNN and its derivative networks deeper and better, [12] employs a residual strategy and skip connections to further the RNN development.

The Transformer based algorithm [4] and its variants [5, 13–16] achieve the most advanced results on multiple language pairs only based on the attention mechanism. Research [13] improves the effect by increasing the scale of model parameters. Still, increasing the number of parameters means that more extensive data sets are needed, and the training is more complicated. It is not suitable for hardware, especially edge devices. CNN-based algorithms [17, 18] are concerned because of their high parallelism. Among them, [17] proposes a CNN-based machine translation algorithm with higher parallelism and a shorter long-term dependency than RNN.

Machine translation projects employ a variety of Transformer structures to optimize the size of the model and precision, as well as the bandwidth required for hardware deployment. Research [2] proposes a pruning algorithm to increase model sparsity and deploy the model on GPU, and research [19] proposes a sparse matrix calculation method. Both of them reduced the bandwidth requirements of matrix calculation on hardware. One is to improve the activation functions such as the ReLU and SoftMax. Research [20] introduces a novel activation function WReLU for lightweight neural network design. Research [16] introduces a random calculation method to replace the traditional SoftMax calculation, which reduces the calculation complexity and improves the speed. Research [14] is a collaborative processing scheme that combines the advantages of multiple networks, and it combines BiLSTM and recurrent attention for machine translation tasks.

These works have effectively promoted the development of machine translation. However, most of the existing Transformer schemes dealing with machine translation tasks only use the attention mechanism and lack the research results combined with the CNN model. Most optimized networks are still too large, and there are defects in input sequence order when using the RNN model to process sequence information. The effect of processing sequence information using the CNN model is not ideal, making them challenging to deploy in edge devices. Based on these, the new activation function Sparse-ReLU, CNN submodel structure, Transformer submodel structure, and cooperation scheme proposed in this research achieve a better effect under a particular parameter scale condition.

### 1.2. Method

**Figure 1:**
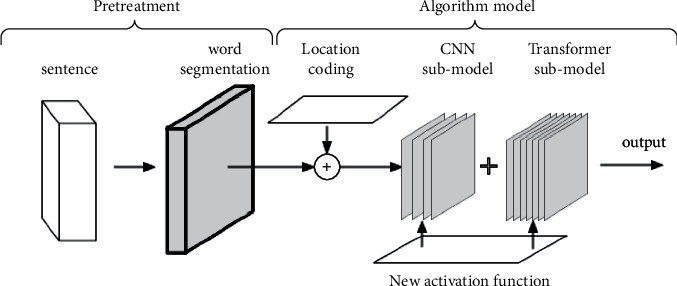
Overall structure of the algorithm.

Unlike prior machine translation Transformer algorithms, this study proposes a Transformer model with few parameters, a CNN submodel, and a novel activation function Sparse-ReLU. CNN and Transformer submodels use Sparse-ReLU to optimize the effect. The three of them cooperate in dealing with machine translation tasks. CNN can process local information of word vectors and extract multiple features containing position-coding, and the attention mechanism can process local features extracted by CNN rather than input sentences.  Figure 1 shows the process of our algorithm.

Figure 1 depicts the translation process. The input and output of the algorithm model are symbol sequences, and the word segmentation operation of the input symbol sequence uses the BPE word segmentation method. The position-coding operation embeds the position information into the symbol sequence obtained by word segmentation. The CNN submodel extracts the features of sentences containing location coding information. The Transformer submodel further extracts the output information of the CNN submodel. Both submodels use Sparse-ReLU.

## 2. Model Structure

### 2.1. Activation Function That Can Improve Sparsity and Accuracy

Most neural network algorithms currently require activation functions to introduce nonlinear operations. However, activation functions such as sigmoid have the disadvantages of complex hardware implementation and high resource consumption in algorithm deployment. When implementing the algorithm in hardware, sparse matrix acceleration is a viable option, and sparse matrix acceleration necessitates high weights sparsity. The ordinary activation function can not maximize sparsity matrix compression technology. Based on this, this research proposes a new activation function Sparse-ReLU for hardware optimization. The activation function has the advantages of low hardware implementation cost and low computing time like the traditional ReLU function. It solves the disadvantages of limited representation ability and insufficient flexibility of the conventional ReLU function.

For machine translation and other applications in natural language processing, real-time computing requirements are high. Because of its low power consumption and small area, the edge devices cannot effectively deploy translation algorithms with huge parameters. In particular, the Transformer algorithm, although the translation effect is good, can only be deployed on the server-side. It is of great significance to realize the activation function with high flexibility and simple hardware implementation.

The activation function designed in this research can improve the sparsity and accuracy of neural networks. The expression is shown in formulas (1)–(4), in which the range of parameter *a* is (0, 1), the content of *b* is (0,1), which satisfies *a* < *b* and *c* < *d*, and both a and *b* need to meet the relationships of 0.125*x*, to complete the multiplication calculation only through the hardware shift operation. As shown in formula (1)–(3), the three subfunctions of Sparse-ReLU are *y*_0_, *y*_1_, and *y*_2_.

(1) $$\begin{array}{c} y_{0}=0, \end{array}$$

(2) $$\begin{array}{c} y_{1}=a\times \left(x-c\right), \end{array}$$

(3) $$\begin{array}{c} y_{2}=b\times \left(x-d\right). \end{array}$$

The function of the subfunction *y*_0_=0 shown in Formula (1) is to set the activation value of the neural network to 0 and improve the sparsity of its activation value. The traditional ReLU function sets the input of all negative numbers to 0 and the positive part to itself. The *y*_0_ function in this research puts the output to 0 no matter what the input is. However, doing so will cause all information to be lost, so formulas (2) and (3) are required to retain the information. Taking the maximum value of the three subfunctions can obtain the formula of Sparse-ReLU.

(4) $$\begin{array}{c} \text{Sparse}-\text{ReLU}=f\left(x\right)=\underset{x\in \left(-\infty ,+\infty \right)}{\text{max}}\left(y_{0},y_{1},y_{2}\right). \end{array}$$

**Figure 2:**
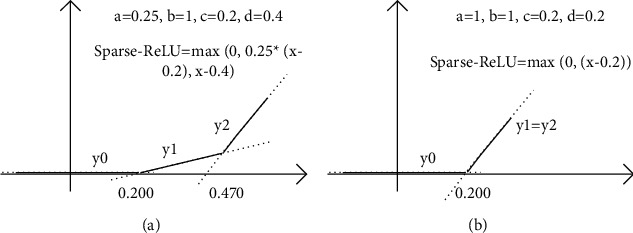
Sparse-ReLU function.

When determining parameters *a*, *b*, *c*, and *d*, the functions with large *c* and *d* are preferred under the same translation effect, improving the sparsity of the weights and activations of neural network. When setting the parameters of Sparse-ReLU to *a* = 0.25, *b* = 1, *c* = 0.2, and *d* = 0.4 in Figure 2(a) , the translation effect of the activation function is the best. According to the formula, the traditional ReLU function is a subset of the activation function designed in this research. Figure 2(b) is the image of setting parameters *a* = 1, *b* = 1, *c* = 0.2, *d* = 0.2 to make *Y*1 = *Y*2 in Sparse-ReLU. In this case, Sparse-ReLU degenerates into an offset traditional ReLU function. Furthermore, ReLU is a subset of Sparse-ReLU, and the characterization power of Sparse-ReLU is higher.

In this research, Sparse-ReLU replaces the traditional function to provide nonlinear characteristics for the network. It is applied between two fully connected layers or between the CNN layer and the fully connected layer in the network. Parameters *a*, *b*, *c*, and *d* in Sparse-ReLU are found through training, to make the effect of the network model better than that of the traditional ReLU activation function. According to the iterative experiment, the German-English translation task of IWSLT14 dataset performs best when the parameters are *a* = 0.25, *b* = 1, *c* = 0.1, and *d* = 0.4. After determining parameters in Sparse-ReLU, combined with the pruning operation, the sparsity of the network model is improved. Unlike other sparsity improvement methods, this activation function can enhance the sparsity of weight parameters and the sparsity of activation, which is convenient for further hardware acceleration using a sparse matrix.

**Figure 3:**
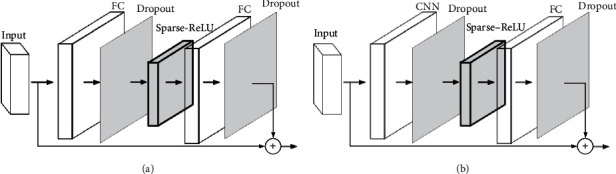
The deployment method of Sparse-ReLU function.

Because it can efficiently use shift and addition operations to realize the activation function in hardware and complete the prediction task of the model based on Sparse-ReLU, Sparse-ReLU can obtain multiple zero values, in which the experimental statistics are more than 50% higher than the value close to zero in the ordinary ReLU activation value. So, it can effectively improve the network sparsity. It has the characteristics of low resource consumption in hardware implementation and can enhance the sparsity of neural network parameters and the accuracy of the model prediction.  Figure 3 shows the application of Sparse-ReLU in the model, as discussed in Section 1.2.

### 2.2. Transformer and CNN Submodels Combined with Sparse-ReLU

Figure 1 shows the overall design, which is made up of a CNN and a Transformer submodel. We will introduce the Transformer structure used in this research in detail.

**Figure 4:**
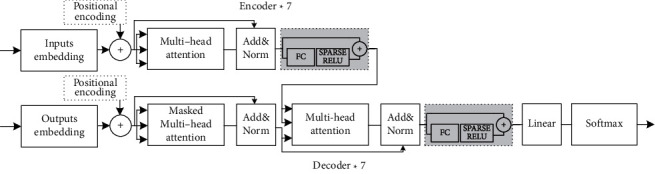
Structure of Transformer submodel.

The Transformer structure has a high ability to extract sequence information.  Figure 4 shows the Transformer structure in this research, composed of the residual layer, multihead attention layer, Sparse-ReLU layer, and layer normalization. As shown in Table 1, the parameters of each Transformer structure are listed. It has the characteristics of low parameter quantity. The Encoder combines Sparse-ReLU, which can improve prediction accuracy.

**Figure 5:**
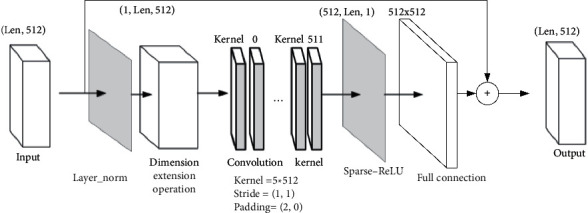
Structure of CNN submodel.

The CNN submodel needs to process the input sentences and extract the features without changing the size of the feature map. Convolution can improve the extraction ability of local features of the network.  Figure 5 shows the CNN submodel. After the sentence is entered into the model, it needs to go through embedding and position-coding operation and then carry out layer normalization operation. Finally, utilizing two-dimensional CNN to process the sentence coding results containing position information.  Figure 5 shows the CNN structure, where Len is the sentence length.

The input channel of CNN is 1, the output channel is the length of the word vector, that is, 512 channels, the size of convolution kernel is (5,512), the stride step is 1, and the size of padding is two zeros for rows and no padding for columns. The dimension of the feature map before CNN processing is (Len, 512), Len is the sentence length, and the dimension of the feature map after CNN is (512, Len). After dimension transformation and Sparse-ReLU, the fully connected layer maps the result to another dimension and makes the residual connection with the original input.  Table 2 lists the detailed measurements of each structure of CNN.

### 2.3. Collaborative Processing Scheme between CNN and Transformer Submodels

**Figure 6:**
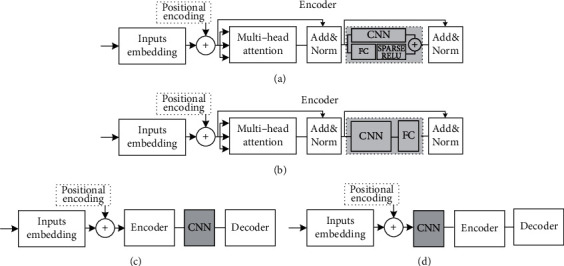
Collaborative scheme of transformer and CNN submodel.

Considering the strong ability of CNN to extract local features, the combination of Transformer and CNN submodels can effectively improve the power of the algorithm to extract local features. There are a variety of cooperative processing strategies for multinetworks, including the concatenation operation method [21], result addition method [22], result point multiplication method [23], and matrix transformation after the concatenation operation. The model in research [21] adopted the method of submodules in series, effectively combined CNN and attention, and proposed an end-to-end ResNet structure model, which was used to extract local features, and summarized the local feature sequence through the attention mechanism. This research discusses the impact of CNN and Transformer on machine translation tasks.  Figure 6 shows the details of various collaborative processing schemes discussed in this research. The CNN submodel of Figure 6 is the structure in Figure 5. Encoder and decoder are also the forms discussed in Figure 4.

**Figure 7:**
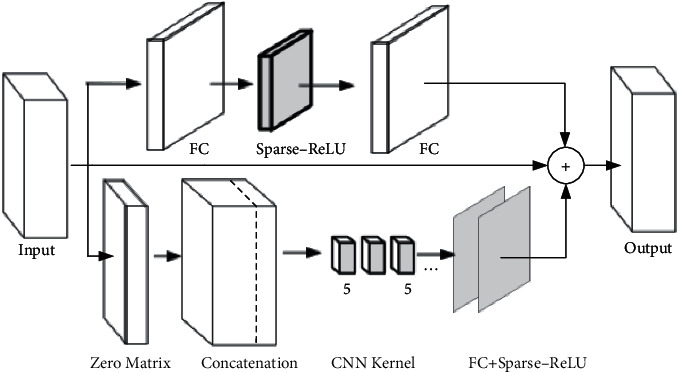
Structural details of CNN in Figure 6(a).

To make the size of the matrix output of the CNN submodel be the same as that of the attention submodel, we set the number of output channels of the convolution kernel to be 512, which is the same as the length of the word vector in the matrix output of attention submodel.  Figure 6(a) shows the scheme that the attention submodel learns the feature of input data, and the feed-forward module consisting of a CNN and a fully connected layer processes the result of attention computation, which is illustrated with the gray box. As shown in Figure 7, the output characteristic matrix of the feed-forward module is the result of the addition of a CNN and a fully connected layer.  Figure 6(b) shows how to add a CNN submodel after the multihead attention layer of the encoder in the Transformer.  Figure 6(c) uses the attention submodel to summarize the feature sequence from the original sentence, then uses a CNN submodel with a ResNet structure to extract local features from the features summarized by the attention submodel, and finally uses the decoder submodel to decode the target text.  Figure 6(d) shows that the CNN submodel first processes the input sentence and learns the local features of the sentence. The Transformer submodel extracts the sequence features processed by the CNN submodel. After the encoder operation in the Transformer, it is handed over to the decoder submodel for decoding operation again.

### 2.4. Algorithm Model

**Figure 8:**
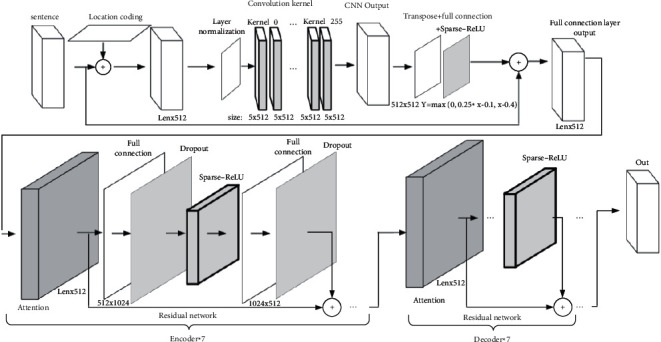
Final translation model combined with Sparse-ReLU.

Figure 8 shows the details of the model in this research. According to the introduction of the previous three sections, the model in this research mainly includes the convolutional neural network feature extraction layer, encoder layer, and decoder layer.  Table 3 shows the structural parameters of the algorithm.

## 3. Experiment and Result Analysis

### 3.1. Machine Translation Dataset and Word Segmentation Algorithm

This research selects the German-English translation task for the experiment, and the dataset is IWSLT14. The training set contains 160250 sentences, and the testing set uses 6750 independent sentences [24].

The input and output of the translation algorithm are symbol sequences. These symbols are the basic units of sentences. Because extensive vocabulary cannot be naturally decomposed into words, using words as the basic units to form sentences will make it challenging to train the algorithm. An alternative is to use word segmentation algorithms such as [19, 25] to learn subwords from the dataset. This research uses the method of research [19] for word segmentation, which introduced the BPE algorithm variant for word segmentation, which can encode the available vocabulary with the vocabulary of variable length subword units. In this experiment, the size of the word table is 10000.

### 3.2. Evaluation Metric

Many automatic evaluation metrics have been proposed in the machine translation task to evaluate the quality of translation results. This research adopts the most popular BLEU [26] evaluation. It summarizes the overlapping words and phrases between machine translation and reference results. The translation results judged by the BLEU evaluation metric are highly consistent with those considered by human beings and have become a de facto translation evaluation standard after being proposed. This research does not use single testing set for BLEU score evaluation but combines multiple testing sets for score evaluation.

### 3.3. Experimental Environment and Model Training

This experiment uses 8 Titan XP graphics cards. PyTorch version is 1.4.0, and the CUDA version is 10.2.  Table 4 lists the detailed configuration used in the experiment.

The algorithm uses a dropout operation to prevent training overfitting to ensure the training quality [27]. We use the dropout operation before the layer normalization of the CNN submodel, multihead attention submodel, encoder and decoder submodel output, and final output layer.  Table 3 lists the network parameter configuration. The step of warmup is 8000. In the prediction process, a beam search algorithm is used instead of a greedy algorithm to obtain better prediction results, where the beam size is 5.

### 3.4. Result Analysis

#### 3.4.1. BLEU Score Comparison

This model is being used to test German-English translation tasks.  Table 5 lists the translation results of this algorithm and expected results.

Table 6 lists the comparison between the translation results of various types of machine translation algorithms and the algorithms in this research. The parameter size of the algorithm proposed in this research is 37.99 M, and the BLUE score reaches 35.24, which is 52.554% higher than other schemes such as research [17], 17.860% higher than research [29], and 2.442% higher than research [16]. The score of the algorithm model in this work is enhanced by 2.323% compared to the classic Transformer model [4], and the parameter size is decreased by 11.28 M, which is reduced by 23%. Compared with Dynamic CNN [30], the score of the proposed algorithm is 1.264% higher, and the parameter size is decreased by 247.01 M, which is reduced by 87%. Compared with the Transformer using the ReLU, the score of the Transformer using Sparse-ReLU is improved by 0.87 scores from 34.29 scores to 35.16 scores, an increase of 2.54%. The score of the optimization result using Sparse-ReLU and CNN is 35.24, an increase of 2.77%.

According to the four cooperation schemes between CNN and Transformer designed in Section 2.3, Table 7 lists the translation results obtained by the seven structures. The CNN structure of structure 2 is the one-dimensional CNN proposed in Figure 7, and the other CNN structures are the two-dimensional CNN submodel structure proposed in Figure 5. The input channel is 1, and the Transformer submodule is the structure proposed in Section 2.2. The input and output channels are word vector lengths, and the word vector dimension is the input channel of the convolution kernel. Using structure 7, when the convolution kernel size is 5 × 512, the BLEU score achieves the best result of 35.24 points.

**Figure 9:**
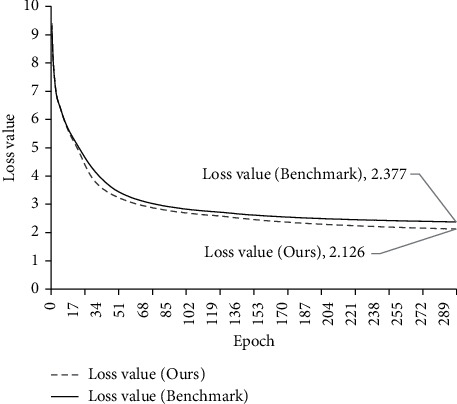
Falling curve of loss value of each structure.

Figure 9 shows the comparison results of the decline curves of loss during different structure training. The solid line in the figure is the loss decline curve of the benchmark model, and the dotted line is the loss decline curve of the Transformer model with CNN and Sparse-ReLU. Compared with the benchmark model, the loss reduction speed of the model with CNN and Sparse-ReLU is much higher than that of the benchmark model. Under the same 300 epochs, the loss value is reduced by 10.6%.

#### 3.4.2. Sparsity Comparison

Table 8 lists the effects of Sparse-ReLU and ReLU on the sparsity of the algorithm. Compared with the conventional ReLU function, the Sparse-ReLU proposed in this research increases the sparsity of the relevant layer by 150.95% and reduces the loss value by 42.2%. Both indexes have an excellent optimization effect.

When the parameters of the new activation function are set to *a* = 0.25, *b* = 1, *c* = 0.2 and *d* = 0.4, the algorithm is pruned to improve the sparsity of the parameters.  Table 9 lists the effects of traditional and new activation functions on the sparsity of the model when the model adopts different pruning algorithms.  Table 9 shows that, compared with L1 norm pruning, the random unstructured pruning algorithm and Sparse-ReLU jointly improve the sparsity of the weight to 78.26% and the sparsity of the activation value of the tested layer to more than 120%.

**Figure 10:**
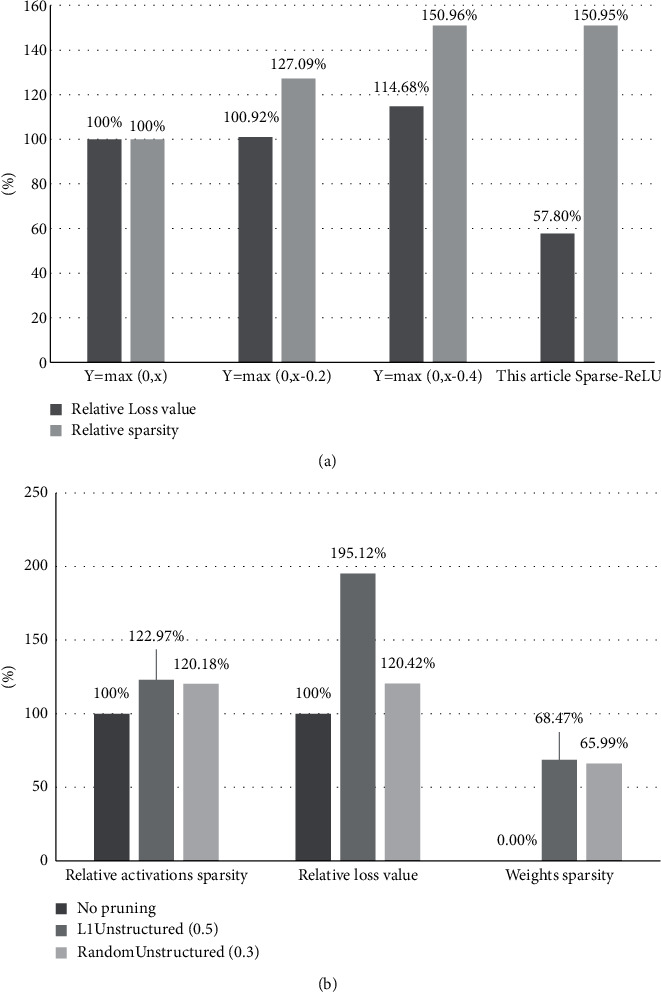
The influence of Sparse-ReLU on sparsity and accuracy.

The bar graph of key information in the above two tables is shown in Figure 10. Figure 10(a) shows that the Sparse-ReLU used in this paper significantly decreases the loss value and improves the sparsity when training the model compared with ReLU. According to Figure 10(b), when the model with Sparse-ReLU uses different pruning algorithms, it can further improve the sparsity with little accuracy loss.

## 4. Conclusion

This paper proposed a cooperative machine translation algorithm based on CNN and Transformer submodels combined with Sparse-ReLU, where the CNN is used to extract local features of sentences containing location information, the Transformer is used to further extract sequence features, and the Sparse-ReLU can optimize the algorithm. Compared with the traditional counterpart, the count of parameters decreased by 23% with accuracy increased by 2.77%, and the sparsity increased by 50%. Consequently, Transformer and CNN parameters are only 36.42 M and 1.57 M, respectively. Test results show that the proposed scheme can effectively improve the accuracy of model translation and the sparsity of activation and weight value.

In future works, the author of this research will continue to study the collaborative scheme between CNN and Transformer and the parameter training method of Sparse-ReLU, hoping to achieve better results.

## Figures and Tables

**Table 1:** Transformer submodel size.

Description	Substructure	Layer name	Size
Encoder	MultiHeadAttention	cast_queries	(512, 384)
		cast_keys_values	(512,768)
		cast_output	(384,512)
		softmax	softmax
		layer_norm	eps = 1e-05
	PositionWiseFCNetwork	LayerNorm	eps = 1e-05
		fc_1	(512,1024)
		fc_2	(1024,512)
		Sparse-ReLU	Sparse-ReLU ：*a* = 0.25,*b* = 1,*c* = 0.2,*d* = 0.4

Decoder	Embedding	Embedding	(10000, 512)
	MultiHeadAttention	tgt_emb	(10000, 512)
	MultiHeadAttention	pos_emb	(10000, 512)
	PositionWiseFCNetwork	Sparse-ReLU	Sparse-ReLU ：*a* = 0.25,*b* = 1,*c* = 0.1,*d* = 0.4

Output	LayerNorm	LayerNorm	eps = 1e-05
	Fc	Fc	(512,10000)

**Table 2:** CNN submodel size.

Description	Substructure	Layer name	Size
Pretreatment	Embedding and location coding	Embedding	(10000, 512)

CNN	Convolution submodel	LayerNorm	eps = 1e-05
		Conv2d	in_ch = 1, out_ch = 512, kernel = (5, 512), stride = (1, 1), pad = (2, 0)
		Linear	(512, 512)
	Activation function	Sparse-ReLU	Sparse-ReLU ：*a* = 0.25,*b* = 1,*c* = 0.1,*d* = 0.4

**Table 3:** Parameters of the model.

Parameter name	Small model
d_model	512
n_heads	3
d_queries	128
d_values	128
d_inner	1024
n_layers	7
max_len	300
cnn_kernel	(5,512)
beam_size	5

**Table 4:** Experimental environment.

CPU	Intel(R) Xeon(R) silver 4116 CPU @ 2.10 GHz
Experiment framework	PyTorch 1.4.0
GPU	8 TITAN XP graphics cards
CUDA	CUDA 10.2
OS	Ubuntu 16.04.12

**Table 5:** Translation results of the model.

Standard results	Results of this research
And of course, we all share the same adaptive imperatives	And of course, we all share the same adaptive applications
We're all born. We all bring our children into the world	We're all born. We bring children to the world
And the great indicator of that, of course, is language loss	And the key indicator for this is the extinction of languages

**Table 6:** The comparison results between this model and others (German-English translation task using IWSLT14 dataset).

		BLEU	Size M	Model
Research [17]		23.1	—	Encoder model based on 6-layer CNN.
Research [28]		28.83	—	Tag-less backtranslation
Research [29]		29.9	—	Linear transformer
Research [16]		34.4	—	Random feature attention
Research [30]		34.8	285	Pay less attention with lightweight CNN
		35.20	296	Pay less attention with dynamic CNN
Traditional transformer model [4]		34.44	49.27	Traditional transformer model
This paper scheme	Small model + Sparse-ReLU	34.29	36.42	Small transformer
		35.16		Sparse-ReLU + Small transformer
	Small model + Sparse-ReLU + CNN	35.24	37.99	Sparse-ReLU + small transformer + CNN

**Table 7:** Comparison of translation results of different structures between CNN and Transformer (512 convolution output channels).

Structural details	Convolution type	Convolution kernel size	BLEU
Structure 1: Figure 6(a)	2d convolution	Same as structure 7	34.32
Structure 2: Figure 6(a)	1d convolution	in_ch = 512, kernel = (5, 1), stride = (1, 0), pad = 0	34.15
Structure 3: Figure 6(b)	2d convolution	in_ch = 1, kernel = (3, 512), stride = (1, 0), pad = (1, 0)	31.46
Structure 4: Figure 6(b)	2d convolution	Same as structure 7	33.61
Structure 5: Figure 6(c)	2d convolution	Same as structure 7	30.86
Structure 6: Figure 6(d)	2d convolution	in_ch = 1, kernel = (7, 512), stride = (1, 0), pad = (3, 0)	34.37
Structure 7: Figure 6(d) (This research adopts)	2d convolution	in_ch = 1, kernel = (5, 512), stride = (1, 0), pad = (2, 0)	35.24

**Table 8:** Influence of Sparse-ReLU on model sparsity.

Sparse-ReLU parameters	Formula	Relative loss value	Relative sparsity
*a* = 1, *b* = 1, *c* = 0, *d* = 0	*Y* = max(0, x)	100% (Baseline)	100% (Baseline)
*a* = 1, *b* = 1, *c* = 0.2, *d* = 0.2	*Y* = max(0, x-0.2)	100.92%	127.09%
*a* = 1, *b* = 1, *c* = 0.4, *d* = 0.4	*Y* = max(0, x-0.4)	114.68%	150.96%
*a* = 0.25, *b* = 1, *c* = 0.2, *d* = 0.4	*Y* = max(0,1/4(x-0.2), x-0.4)	57.80%	150.95%

**Table 9:** Experimental results of the combination of Sparse-ReLU and pruning algorithm.

Pruning algorithm	Set parameters	Relative activations sparsity	Relative loss value	Weights sparsity
No pruning + Sparse-ReLU	\	100% (Baseline)	100% (Baseline)	0.00% (Baseline)

L1Unstructured + Sparse-ReLU	0.3	121.22%	173.60%	22.58%
	0.5	122.97%	195.12%	68.47%

RandomUnstructured + Sparse-ReLU	0.3	120.18%	120.42%	65.99%
	0.2	120.55%	110.79%	48.70%
	0.4	120.70%	288.05%	78.26%

## Data Availability

The experimental data used to support the findings of this study are included within the article. The dataset data used to support the findings of this study are available from the corresponding author upon request.
